# Insights into the proteomic profile of newly harvested corn and metagenomic analysis of the broiler intestinal microbiota

**DOI:** 10.1186/s40104-021-00656-1

**Published:** 2022-02-09

**Authors:** Dafei Yin, Youli Wang, Liqun Wang, Yuqin Wu, Xiaoyi Bian, Samuel E. Aggrey, Jianmin Yuan

**Affiliations:** 1 College of Animal Husbandry and Veterinary Medicine, Shenyang Agricultural University, Shenyang, 110866 China; 2 State Key Laboratory of Animal Nutrition, College of Animal Science and Technology, China Agricultural University, 100193 Beijing, China; 3 NutriGenomics Laboratory, Department of Poultry Science, University of Georgia, Athens, GA 30602 USA

**Keywords:** Broilers, Corn, Metagenome, Proteome, Storage

## Abstract

**Background:**

The use of newly harvested corn in feed causes wet droppings in broilers and increased feed cost which was termed as “new season grain problem”. The present study was conducted to evaluate the proteomic profile of newly harvested corn and the subsequent influence on intestinal microbiol community for broiler chickens.

**Methods:**

Newly harvested corn stored for either half a month (HM) or two months (TM) was used, and the pasting properties, total soluble sugars, and proteomic analysis technology was used to explore the influence of storage on natural aging corn properties. Additionally, seventy-two 7-day-old Ross 308 male broiler chicken were fed with different stored corn. Apparent metabolizable energy (AME), digesta viscosity, intestinal morphology and microbiota were examined to explore the influence of feed corn storage on broiler chickens.

**Results:**

Pasting properties in the TM corn exhibited decreased viscoelastic properties. Proteomic studies found a total of 26 proteins that were differentially expressed between the two treatment groups. Proteins involved in starch and polysaccharides biosynthesis were upregulated in TM compared with HM. Chickens fed on TM diet had higher relative energy utilization compared to the HM birds. With increased corn storage, the relative digesta viscosity decreased significantly (*P* ≤ 0.05). The total number of goblet cells and lymphocytes was lower in chickens fed the TM diet. The microbiota data showed that the TM chickens had decreased abundance of diarrheal bacteria such as *Hungatella hathewayi* and *Bacteroides fragilis*, and increased butyrate-producing bacteria such as *Alistipes* compared to the HM chickens.

**Conclusions:**

Storage of newly harvested corn induced the synthetic reaction of large molecules and changed the solubility of starch and protein with increasing soluble sugars and decreasing pasting properties that may improve the fermentation of intestinal microbiota, improve the energy utilization and protect gut health without the risk of diarrhea.

**Supplementary Information:**

The online version contains supplementary material available at 10.1186/s40104-021-00656-1.

## Introduction

Corn is the most abundantly grown cereal grain and has high nutritional value, especially for poultry and swine. As corn production is seasonal, corn grains need to be stored throughout the year to provide sufficient supplies for feed industries. However, there is an asynchrony of supply and demand that leads to a large number of newly harvested corn being processed as animal feed during each harvest season. The use of newly harvested corn in poultry diets is especially problematic because it decreases the apparent metabolizable energy (AME) and feed conversion ratio and causes wet droppings and increased feed cost [1–3]. Nutritionists have termed this problem the “new season grain problem” [4].

Newly harvested grain should be stored for a period of several weeks or months to enhance its nutritional value [2]. During storage, respiratory and metabolic events continue, and changes in internal factors, such as starch, endogenous enzymes and carbonyl compounds in cell walls and external factors occur [5, 6]. Among these factors, endogenous proteolytic enzymes play a central role in regulating the synthesis and decomposition of carbohydrates and proteins [7, 8].

Proteomics is an important tool that can be used to determine the biological roles and functions of individual proteins that govern grain seed quality and allow for the systematic analysis of complex cellular mechanisms [9]. Reports of corn proteome expression during the artificial aging period indicate that artificial aging would increase proteases and breakdown stored proteins, and impair metabolism and energy supplies [10, 11]. However, the physicochemical properties of naturally aging corn have yet to be elucidated. Our objective was to delineate the proteomic mechanisms that underlie differences between corn stored for different time.

There is a clear link between bird performance and gut microbiota composition [12]. The intestinal microbiota plays an important role in maintaining normal gut function and contributes to the development of functional gastrointestinal symptoms by modulating the signaling pathways of hosts [13]. We have previously shown that using newly harvested corn leads to overfeeding and increased ileum lesions and injuries in broiler chickens [3]. Intestinal injury is associated with diarrhea and impaired gut resistance to pathogens [14]. Leonard et al. [15] reported that grain (rice, oats, barley and corn) protein is one of the most common triggers of enterocolitis syndrome. Additionally, non-digestible polysaccharides in corn can be broken down by members of the intestinal microbiota, producing monosaccharides and short-chain fatty acids (SCFAs), leading to unbalanced energy status [16]. Intestinal bacterial composition changes and their subsequent metabolic changes when a diet containing newly harvested corn is fed to broiler chickens and how they affect performance and health are unclear.

We herein first employed a combined high-throughput label-free comparative proteomics and metagenomics to study the biological mechanisms that underlie the relationship between the composition of natural aging corn and the diversity of the complex intestinal microbial community of broiler chickens.

## Materials and methods

### Plant material

One newly harvested mixed corn variety corn (ZhengDan 958) was sourced from Hebei Province. The corn was sown in March 2015 and harvested in September 2015. After natural drying to 14% moisture content, intact corn grains were stored from September to November in a warehouse located at the China Agricultural University Poultry Experimental Base without air conditioning to simulate typical grain storage conditions. The newly harvested corn was sampled after storage for half a month (HM) or two months (TM), frozen immediately in liquid nitrogen and stored at − 80 °C until further analysis. At the same time, broilers fed the corn stored for different period of the time were used to further examine the differences between different storage times of corn. To eliminate the influence of the environment and animals, the same variety of corn stored for one year with the same moisture content was treated as the control in the broiler trial.

### Determination of pasting properties, total soluble sugar, glucose and fructose

The corn samples were analyzed for pasting properties and soluble sugar. The pasting properties of corn mixtures stored for different times and suspended in distilled water were determined by a Rapid Visco-Analyzer (model RVA-4C, Newport Scientific Pty. Ltd., Warriewood, Australia) and estimated by the method of Achayuthakan and Suphantharika [17]. Total soluble sugars were estimated by the phenol-sulfuric acid method of Dubois et al. [18], using sucrose as the standard. Glucose and fructose were determined by the HPLC method of Wilson et al. [19].

### Label-free proteomic analysis process

#### Protein extraction and digestion

The HM and TM samples were thawed at − 80 °C and then ground and hereafter, 100 mg of each sample was weighed. Then 400 μL SDT lysis buffer (4% SDS, 100 mmol/L Tris-Hcl, 1 mmol/L DTT, pH 7.6) was added to the sample, followed by solubilization for 20s with a tissue homogenizer; this was repeated 5 times. After 20 min ultrasonic treatment, the mixture was centrifuged for 30 min at 10,000 × *g* at 4 °C, and the supernatant was saved for subsequent experiments. The BCA assay was used for protein quantification.

Protein digestion (200 μL for each sample) was performed using the FASP procedure described by Wisniewski [20]. Briefly, the detergent DTT, and other low molecular weight components were removed using 200 μL UA buffer (8 mol/L Urea, 150 mmol/L Tris-HCl, pH 8.0) and facilitated by centrifugation (14,000 × *g* × 15 min). Then, the 100 μL IAA (50 mmol/L IAA in UA) was added in UA buffer, and the mixture was oscillated for 1 min at 600 r/min and allowed to settle for 30 min in the dark at room temperature. The filter was washed with UA buffer (100 μL) and 100 μL of NH_4_HCO_3_ buffer twice. Subsequently, 40 μL trypsin buffer (4 μL Trypsin in 40 μL NH_4_HCO_3_) was added. The mixture was oscillated for 1 min at 600 r/min and allowed to settle for 16–18 h at 37 °C, and the resulting peptides were collected. The filtrate was desalinized with C18 SD Extraction Disk Cartridge and quantified at OD280.

#### LC-MS/MS analysis

Approximately 2 μL product of protein digestion was used for LC-MS/MS analysis and separated using a nanoliter HPLC Ultimate 3000 system (Thermo Fisher Scientific, Louis, MO, USA). The mobile phase A was 0.1% formic acid solution, and the mobile phase B was 0.1% formic acid solution with 80% acetonitrile. The chromatographic column C18 trap column (C18 3 μm 0.10 × 20 mm) was balanced with 95% A solution. The sample was loaded onto the C18 trap column through an automatic sampler and separated by a chromatographic column (C18 1.9 μm 0.15 × 120 mm) at the flow rate of 600 nL/min. The gradient elution procedure was as described in Additional file 1: Table S1

#### Maxquant label-free quantification analysis

The 6 resulting raw LC-MS/MS files were imported to the MaxQuant software (1.3.0.5) and Proteome Discoverer 2.0 (Thermo Fisher Scientific, Bremen, Germany) for database inquiry and LFQ label-free quantification analysis. The database was corn_160605.fasta; enzyme:trypsin; max missed cleavage: 2; fixed modifications: carbamidomethyl (C); variable modifications: oxidation(M), acetyl (Protein N-term); peptide Mass Tolerance: ±15 ppm; Fragment mass tolerance: 20 mm; peptide confidence: high; peptide length: > 4. The cutoff for the global false discovery rate (FDR) in peptide and protein identification was 0.01. Label-free quantification was performed using MaxQuant as previously described [21]. Intensity-based absolute quantification (iBAQ) in MaxQuant was performed to quantify protein abundance for the identified peptides. An FDR estimation for differentially expressed proteins was performed using a mixture model-based method [22]. The significance of the differentially expressed proteins between the samples was examined with the cutoff values *P* ≤ 0.05 and FDR ≤ 0.05.

#### Bioinformatics analysis

Differentially expressed proteins (DEPs) were subjected to Core Expression Analysis. In this study, the DEPs between HM and TM was undertaken. The ratio values in the datasets were converted to fold change values, where the negative inverse was taken for values between 0 and 1. The sequence data for the selected differentially expressed proteins were retrieved from the UniProtKB database in batches and FASTA format. Following the annotation and annotation augmentation steps, the studied proteins were blasted against KEGG GENES (plants) to retrieve the KOs and were subsequently mapped to pathways in KEGG.

### Animals and experimental design

The experiment was assigned to two periods following the corn storage time. During each period, seventy-two 7-day-old male broiler chickens (Ross 308) were obtained from a commercial hatchery and grown for 14 d. In total, 144 Ross 308 male birds were selected. Birds were randomly assigned to one of two dietary treatments (HM vs control or TM vs control) (during each time thirty-six birds fed with control corn, this process repeated twice). There were 6 replicates per treatment and 6 birds per replicate. Feed (pelleted) and water were provided ad libitum. The light regimen was 23 L:1D and the room temperature was 28–30 °C. All the corn samples used to formulate basal broiler diets that met broiler recommendations of NY/T33–2004 (Table S2).

### Sample collection and DNA extraction

**Fig. 1:**
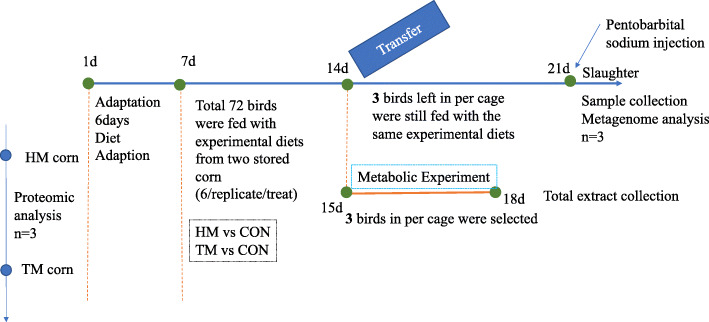
Experimental layout. Seventy two birds were divided into 2 groups, namely, HM and TM. On d 14, half birds in per treatment were selected to do metabolic experiment for three days. The left parts were still fed with experimental diets. On d 21, non-metabolic birds were killed to get samples for metagenome analysis (*n* = 3)

The experimental layout is shown in Fig. 1. At 14 days of age, a metabolic experiment was conducted, and 3 randomly selected chickens (remaining birds were still fed with basal diets) per cage were moved to the new cages and fed with either HM, TM or control metabolic diets (Table S3) for 3 d. Total excreta outputs and feed intakes were recorded from 15 to 18-day post hatching. Excreta were dried in a forced-air oven at 60 °C for 48 h and the gross energy of excreta and metabolic diets were determined using an adiabatic bomb calorimeter to determine apparent metabolizable energy (AME). Feed and extract nitrogen contents were determined by a macro Kjeldahl method to get nitrogen retention for the calculation of nitrogen-corrected AME (AME_n_). The calculation equations are as follows:

$$ {\mathrm{AME}}_{\mathrm{diet}}\left(\mathrm{MJ}/\mathrm{kg}\right)=\left(\mathrm{Gross}\ \mathrm{energy}\ \mathrm{intake}-\mathrm{Gross}\ \mathrm{energy}\ \mathrm{excretion}\right)/\mathrm{Feed}\ \mathrm{intake} $$

$$ {\mathrm{AMEn}}_{\mathrm{diet}}\left(\mathrm{MJ}/\mathrm{kg}\right)=\mathrm{AME}-34.99\times \left(\mathrm{Nitrogen}\ \mathrm{intake}-\mathrm{Nitrogen}\ \mathrm{excretion}\right)/1000 $$

At 21-day post-hatching, the 3 remaining birds in each cage were euthanized by injection with 5% pentobarbital sodium. Immediately following euthanasia, the abdominal cavity was opened and the duodenal contents, approximately 3 cm lengths of duodenum were removed for gut morphological measurements. For viscosity measurements, jejunal digesta were collected and diluted with (1:1) distilled water and homogenized for 20 min at room temperature before centrifugation. The viscosity was measured according to the method of Piel et al. [23].

The cecum was collected within 5 min of euthanasia, immediately placed in cryogenic vials, snap-frozen in liquid nitrogen and stored at − 80 °C until DNA extraction. Total genomic DNA was isolated from 220 mg of frozen cecal contents using the QIAmp DNA Stoll Kit (Qiagen GmbH, Hilden, Germany). The DNA concentration and purity were determined using a NanoDrop 2000 spectrophotometer (Thermo Fisher Scientific, Wilmington, DE, USA). Three samples per treatment were selected for further analysis.

### Morphological examination

Intestinal samples were immersed in formaldehyde, before being fixed in Bouin’s solution and embedded in paraffin. The length of the intestinal villi and the depth of the intestinal crypt were measured with a linear scaled graticule. The number of goblet cells and lymphocytes /μm^2^ area of the villus and crypts was measured by 25 squared graticules.

### Metagenome analysis process

#### DNA library construction and sequencing

DNA libraries were generated using NEBNext® Ultra TM DNA Library Prep Kit for Illumina (NEB, USA) following manufacturer’s recommendations. Briefly, the DNA sample was fragmented by sonication to a size of 350 bp, then end-polished, A-tailed, and ligated with the full-length adaptor for Illumina sequencing. PCR products were purified (AMPure XP system) and libraries were analysed for size distribution by Agilent2100 Bioanalyzer and quantified using real-time PCR. The clustering of the index-coded samples was performed on a cBot Cluster Generation System. Then, the library preparations were sequenced on an Illumina HiSeq platform and paired-end reads were generated.

#### Gene catalogue construction

We performed de novo assembly and gene prediction for the high quality reads of 6 samples in stage I using SOAP denovo v1.06 [24] and GeneMark v2.7 [25], respectively. All predicted genes were aligned pairwise using BLAT and genes, of which over 90% of their length can be aligned to another one with more than 95% identity (no gaps allowed), were removed as redundancies, resulting in a non-redundant gene catalogue comprising of 659,733 genes. This gene catalogue from our cecal samples was further combined with the previously constructed CD-HIT gene catalogue [26], by removing redundancies in the same manner. At last, we obtained an updated gene catalogue that contains 362,337 genes.

#### Taxonomic assignment of genes

Taxonomic assignment of the predicted genes was performed using DIAMOND [27]. In our analysis, we collected the reference microbial genomes from Non-Redundant (NR) of NCBI (v 2014-10-19), and then aligned all genes onto the reference genomes. For each gene, the highest scoring hit(s) above these two thresholds was chosen for the genus assignment. For the taxonomic assignment at the phylum level, the 65% identity was used instead.

#### Functional annotation

The functional annotation and abundance analysis of KEGG (Kyoto Encyclopedia of Genes and Genomes), and carbohydrate enzyme (CAZy) (version: 2014.11.25) were performed using BLASTP (e-value ≤ 1e-5). Based on the species abundance and functional abundance, the abundance cluster analysis, and sample cluster analysis were performed.

### Statistical analysis

LEfSe analysis uses the Kruskal-Wallis rank sum test to detect significantly different abundances and performs LDA scores to estimate the effect size (threshold: > 2). Statistical analyses were performed using SPSS software, version 16.0 (SPSS Inc., Chicago, IL, USA). The data were subjected to an analysis of variance (ANOVA), the means were compared using Student’s t-tests, and the differences were considered significant at *P* ≤ 0.05.

## Results

### Corn properties

**Fig. 2:**
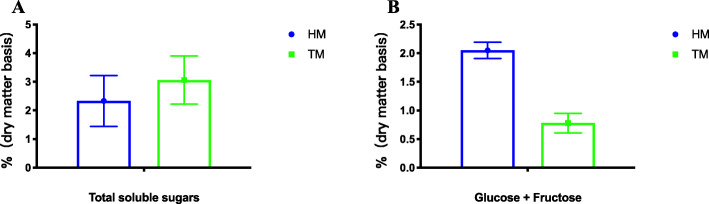
Storage effects on total soluble sugars (A) and total concentrations of glucose and fructose (B) of corn (mean ± SD, triplicate samples). (HM, half month stored corn; TM, two months stored corn)

The pasting characteristics of corn stored at different times determined by the RVA are summarized in Table 1. Storage time resulted in significant (*P* ≤ 0.05) decreases in peak viscosity, final viscosity, and setback viscosity and an increase in pasting temperature. At the same time, a significant change in soluble sugars was observed during storage of corn for the two periods (Fig. 2A). Lower total concentrations (*P* ≤ 0.05) of glucose and fructose were found in corn stored for two months (Fig. 2B).

**Table 1:** Pasting properties of different storage time corn

Sample	Peak viscosity RVA	Breakdown RVA	Final viscosity RVA	Setback RVA	Pasting temperature, °C
HM	1796 ± 42.2	166 ± 21.4	3087 ± 123.0	1457 ± 71.6	77.6 ± 0.13
TM	1602 ± 68.6	99 ± 32.9	2711 ± 34.5	1208 ± 10.6	78.1 ± 0.13
*P*-value	0.014	0.162	0.007	0.004	0.036

Mean ± standard deviation

*HM*, half months stored corn; *TM*, two months stored corn

### Proteome variation of corn in response to different storage times

By merging all identified protein lists including those from two time points and three biological replicates, 2499 proteins were identified and 1984 proteins were quantified from all samples. For further analysis, the TM/HM ratios were log_2_ transformed. Relative quantifications of 1984 proteins were divided into two categories: proteins with quantitative ratios over 1.5 and *P* ≤ 0.05 were considered upregulated, while proteins with quantitative ratios ≤1/1.5 (0.67) and *P* ≤ 0.05 were considered downregulated. Venn diagram analysis showed that 222 proteins were influenced by storage time, among these proteins (Fig. S1).

### Differentially expressed proteins in corn stored for different times

We identified a total of 26 proteins that were differentially expressed, among which only 2 diminished proportionally to HM corn, while 24 proteins seemed to be positively influenced by the increase in storage time (Table 2). Peptidyl-prolylcis-transisomerase and jasmonate-induced protein were downregulated in TM compared with HM. The other identified proteins were upregulated in TM compared with HM. Among these proteins, GRMZM2G162359_P01, GRMZM2G008216_P02 and GRMZM5G825695_P01 proteins have no known functions and no annotations could be found to assign or predict their functions.

**Table 2:** Proteins identified as differentially expressed from Label-free LC-MS/MS

Accession number	Protein description	TM/HM ratio	*P*-value	KO
GRMZM5G866758_P02	Acetyl-CoA acetyltransferase, cytosolic	1.75	0.002	
GRMZM2G174883_P01	Legumin 1	1.67	0.012	K03671
GRMZM2G055434_P01	Early nodulin	1.94	0.013	
GRMZM2G023347_P01	Prefoldin subunit	1.50	0.015	
GRMZM2G010762_P01	Early nodulin-like protein	1.54	0.018	K00700
GRMZM2G020423_P01	Jasmonate-induced protein	0.61	0.019	K00626
GRMZM2G016958_P01	IAA-amino acid hydrolase	2.07	0.019	K01507
GRMZM2G041881_P01	Nascent polypeptide-associated complex subunit beta	4.27	0.020	
GRMZM2G059353_P01	Non-green plastid inner envelope membrane protein	1.81	0.021	
GRMZM2G162359_P01	Uncharacterized protein	1.97	0.021	K13448
GRMZM2G473463_P01	Mitochondrial import inner membrane translocase subunit	1.57	0.026	
GRMZM2G105712_P05	60S acidic ribosomal protein	1.81	0.029	K07304
GRMZM2G397044_P02	Peptidyl-prolyl cis-trans isomerase	0.34	0.031	
GRMZM2G163406_P01	Dirigent protein	2.45	0.033	
GRMZM2G032628_P01	1,4-alpha-glucan-branching enzyme 2	1.95	0.034	K03232
GRMZM2G097457_P01	Pyruvate, phosphate dikinase	1.56	0.036	
GRMZM2G164714_P02	Phosphoenolpyruvate carboxylase family protein	1.57	0.037	
GRMZM2G071433_P01	Plasma membrane associated protein	1.60	0.038	K03016
GRMZM2G008216_P02	Uncharacterized protein	1.67	0.040	
GRMZM2G445905_P03	Cellulose synthase	1.62	0.045	
GRMZM5G825695_P01	Uncharacterized protein	1.85	0.045	K10999
GRMZM2G439201_P02	Elongation factor 1-beta 2	2.09	0.045	
GRMZM2G026470_P01	Soluble inorganic pyrophosphatase 1	1.54	0.046	K01006
GRMZM2G306345_P01	Pyruvate orthophosphate dikinase1	1.61	0.047	
GRMZM2G085260_P01	Desiccation-related protein	1.68	0.049	K02943
GRMZM2G046520_P01	Dirigent protein	2.18	0.050	K01703

*HM*, half months stored corn; *TM*, two months stored corn

### Functional annotation of identified proteins

**Fig. 3:**
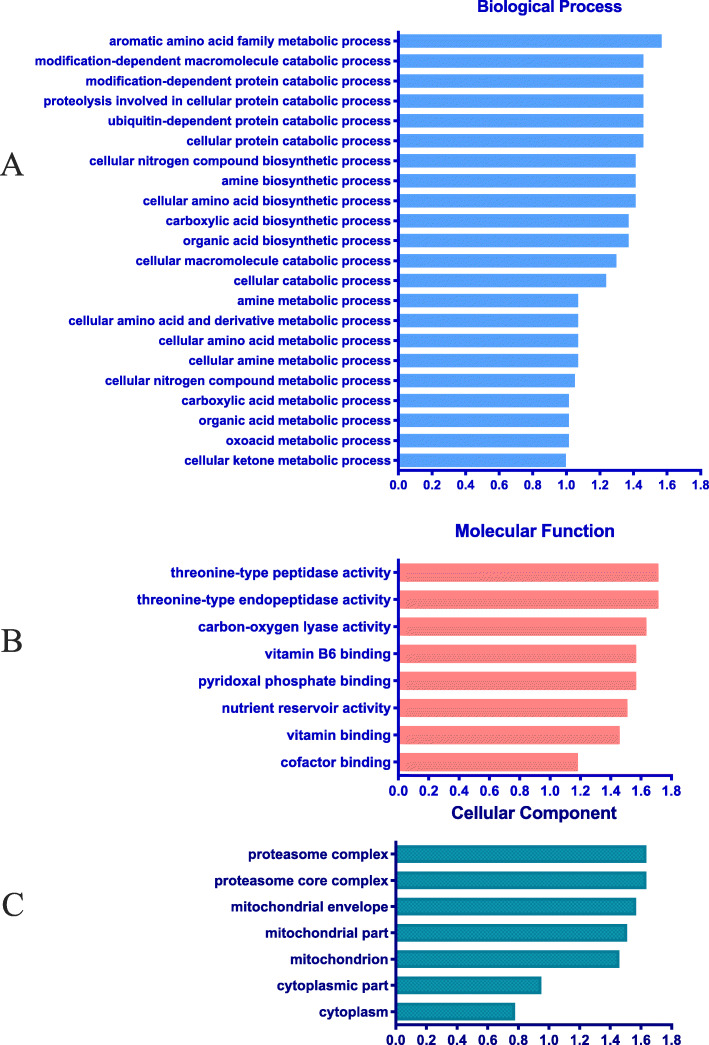
The proportion of the differentially expressed proteins categorized by function. A: Gene Ontology analysis for biological processes; B: Gene Ontology analysis for molecular function; C: Gene Ontology analysis for molecular function

GO terms were assigned to the 222 differentially expressed proteins. Figure 3 shows GO annotations for proteins that were differentially expressed in HM and TM. The identified proteins cover a wide range of cellular processes, molecular functions and cellular components and can be classified into 22, 8, and 7 categories in these broad groups, respectively. The differentially expressed proteins in the biological functions category were mainly associated with cellular ketone metabolic, carboxylic acid metabolic and cellular amino acid biosynthetic processes. The largest group within the molecular function category was cofactor binding. The cellular component functions mainly belong to the cytoplasm and mitochondrion. KEGG results showed for the most differentially expressed proteins suggest that these proteins are involved in 13 pathways (Fig. S2).

### Bird properties affected by corn storage

**Fig. 4:**
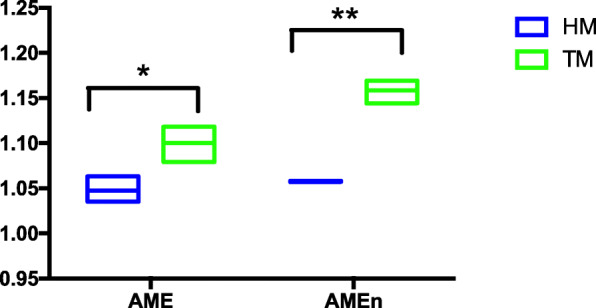
Storage effects on relative AME and AMEn to control groups of 17 d broiler chickens (*n* = 6). (* *P* ≤ 0.05, ** *P* ≤ 0.01. HM, half month stored corn; TM, two months stored corn)

The relative AME and AMEn values of each batch are shown in Fig. 4. Both AME and AMEn were significantly higher (*P* ≤ 0.05) in HM than in TM.

**Fig. 5:**
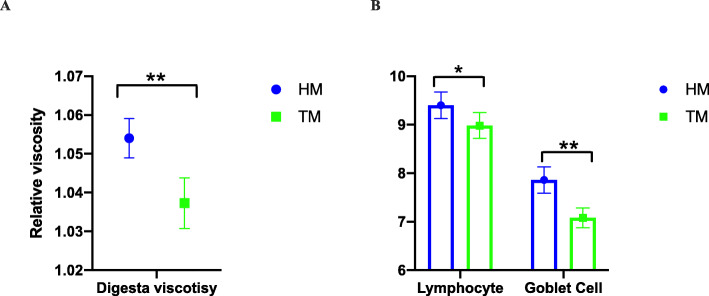
Storage effects on relative digesta viscosity (A) and lymphocytes and goblet cells number in duodenum (*n* = 6). (* *P* ≤ 0.05, ** *P* ≤ 0.01. HM, half month stored corn; TM, two months stored corn)

With increased corn storage, the relative digesta viscosity decreased significantly (*P* ≤ 0.05) (Fig. 5A). The total number of goblet cells and lymphocytes was lower in chickens fed the TM diet (Fig. 5B). There were no significant differences between treatments in crypt and villi (Fig. S3).

### Taxonomic characterization of the gut microbiota

A total of 1,870,386 ORFs were predicted. Among these ORFs, 82.06%, 78.41%, 71.55%, 69.39%, 53.90%, 49.20% and 41.02% had annotations at the kingdom, phylum, class, order, family, genus and species levels, respectively. Unclassified ORFs accounted for 17.94% of unigenes, representing novel rhizosphere taxa. The abundance was compared between groups, number of significantly changed genes are showed in a Venn diagram. When compared to HM corn treatment, a total of 97,141 genes were significantly changed in the TM groups, and 110,678 genes were changed in the HM groups (Fig. S4).

**Fig. 6:**
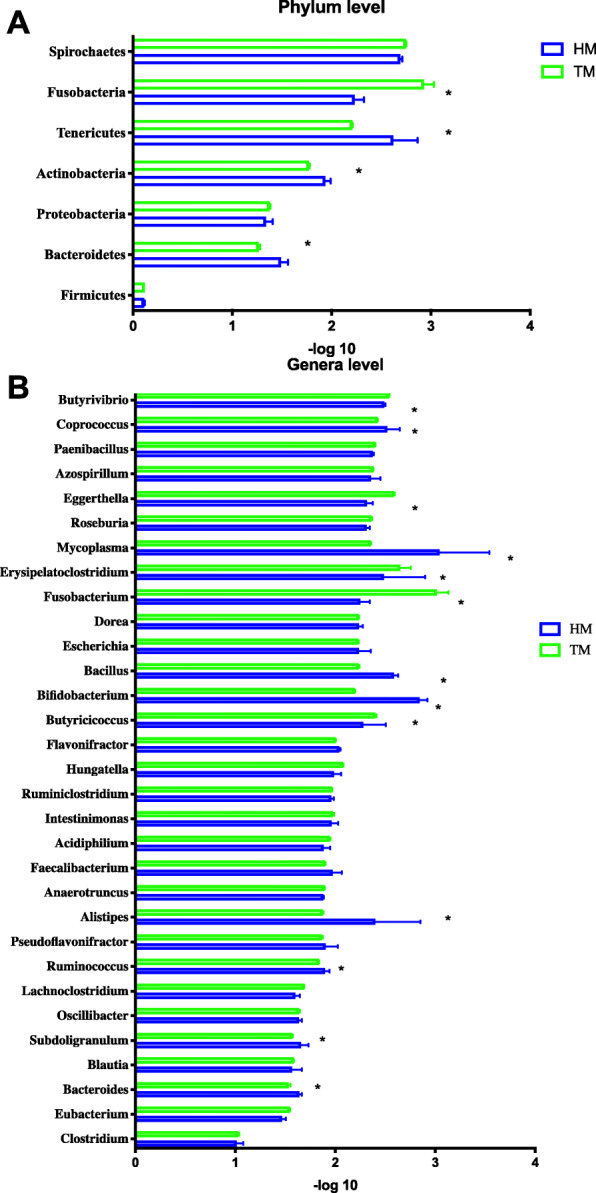
Relative abundance of annotated phylum (A) and significant different genus (B) in cecal microbiota of broilers. (HM, half month stored corn; TM, two months stored corn) * *P* ≤ 0.05

We aligned the reads to a catalog, and the majority of aligned reads were bacterial and dominated by the phyla Firmicutes, Bacteroidetes, and Proteobacteria followed by Actinobacteria, Tenericutes, Fusobacteria and Spirochaetes. Bacteroidetes, Proteobacteria and Tenericutes were significantly enriched in stored corn subjects, while the relative abundance of Fusobacteria was significantly increased in newly harvested corn (*P* ≤ 0.05) (Fig. 6A). *Clostridium*, *Eubacterium*, *Bacteroides* and *Blatutia* were the most abundant genera in our treatments. Compared with the newly harvested corn, stored corn significantly increased 8 kinds of genera bacterium such as *Allstipes*, *Butyricicoccu*s and *Mycoplasma* (Fig. 6B). Species and genome level abundances were also calculated to determine the composition of the gut microbiota (Fig. S5), we compared the composition HM and TM communities and observed increases in *Faecalibacterium prausnitzii*, *Barnesiella intestinihominis*, *Bacteroides dorei* and *Firmicutes bacterium* CAG:475 in TM compared with HM. There were decreases in the abundance of *Bacteroides fragilis*, *Subdoligranulum variabile*, *Ruminococcus torques*, *Eubacterium* sp. ER2, *Clostridium* sp. CAG:678 and *Ruminococcaceae bacterium* D16 in TM compared with HM.

### AME and AMEn are correlated with microbiota

**Fig. 7:**
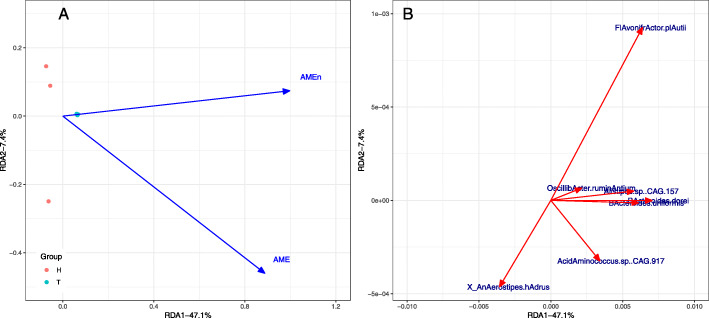
Bacterial abundance RDA correlation biplots by selected explanatory variables. The sites and explanatory variables (A) and species (B) plots are presented separately for clarity; they are divided from the same RDA model. H: half moths storage corn; T: two months storage corn

The global RDA model that selected AME and AMEn as the meaningful explanatory variables was significant (*P* ≤ 0.05) (Fig. 7). The overall variation in species composition was attributed to these explanatory variables, of which the majority were explained by the first and second axes variation, which accounted for 47.1% and 7.4%, respectively. In Fig. 7B, we highlight 7 species that were significantly correlated with one or both of the first two RDA axes. Among these, *Acidaminococcus* sp. CAG.917 was strongly associated with AME while *Alistipes* sp. CAG.157, *Bacteroides dorei*, *Bacteroides uniformis* and *Oscillibacter ruminantium* were correlated with AMEn.

### LEfSe analysis

**Fig. 8:**
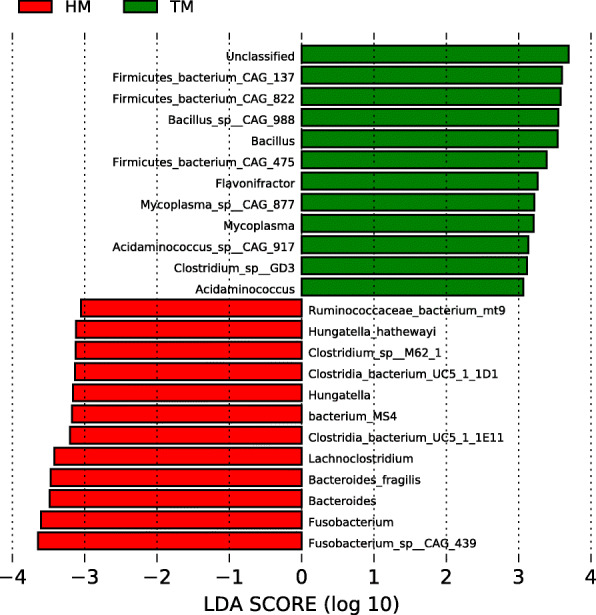
Species differentially represented between HM and TM samples identified by linear discriminant analysis coupled with effect size (LEfSe) (LDA > 2, *P* < 0.05). (HM (green), half month stored corn; TM (red), two months stored corn)

The LEfSe test detected differences in the relative abundances of bacterial taxa across samples (Fig. 8). Among the genes from the species level, *Alistipes* and *Firmicutes bacterium*, *Bacillus*, *Mycoplasma*, *Alistipes inops*, *Barnesiella intestinihominis*, *Barnesiella* and *Ruminococcus* were significantly enriched in the TM treatment, while *Eubacterium desmoians*, *Hungatella hathewayi*, *Hungatella*, *Lachnociostridum*, *Bacteroides fragilis*, *Fusobacterium* and *Eubacterium* were significantly enriched in the HM treatment (LDA > 2, *P* < 0.05).

### Functional analysis and bacterial metabolic processes

**Fig. 9:**
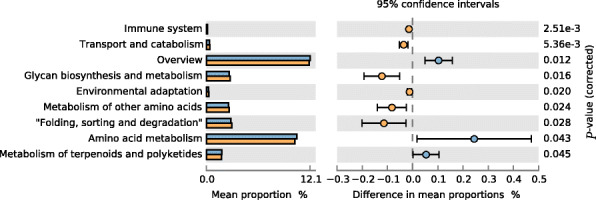
The significant different functions of the cecal microbiota of the broilers. Statistics of the number of annotated genes at KEGG metabolic pathway level two. (HM, half month stored corn; TM, two months stored corn)

**Fig. 10:**
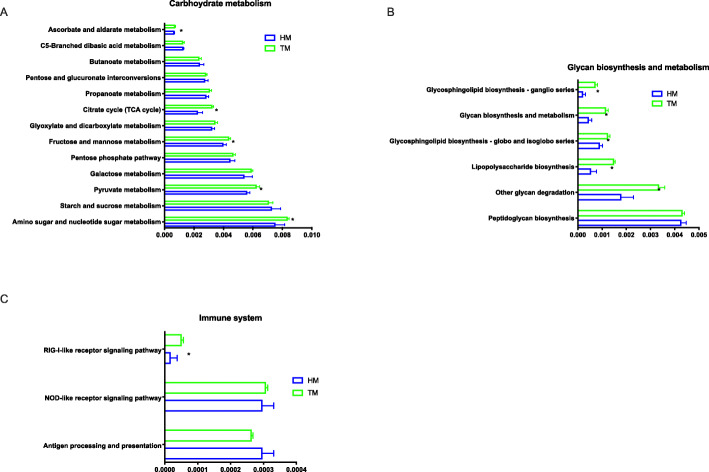
Comparison of gene pathways of the cecal microbiota of broilers annotated genes at KEGG pathways at level three of carbohydrate metabolism (A), glycan biosynthesis and metabolism (B) and immune system (C). (HM, half month stored corn; TM, two months stored corn). * *P* ≤ 0.05

**Fig. 11:**
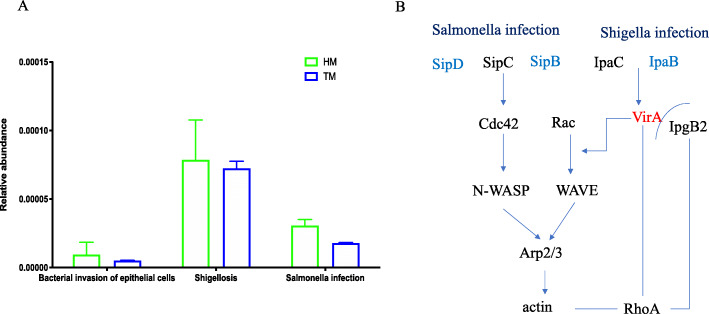
Comparison of pathogenic invasion related pathways of the cecal microbiota of broilers annotated genes at KEGG pathways at level three. (HM, half month stored corn; TM, two months stored corn)

The function of bacterial assemblages associated with different storage times of corn was cataloged using level 3 KEGG orthologs. Several KEGG level 2 modules were different between the two treatments. HM treatment significantly improved the metabolism of terpenoids and polyketides and amino acid metabolism, while TM had beneficially influenced metabolism of other amino acids, folding, sorting and degradation, glycan biosynthesis and metabolism and transport and catabolism and immune system (*P* ≤ 0.05, Fig. 9). At KEGG level 3, compared with HM treatment, TM was enriched for carbohydrate metabolism (e.g., amino sugar and nucleotide sugar metabolism and pyruvate metabolism) (Fig. 10A), glycan biosynthesis and metabolism (e.g., glycan degradation) (Fig. 10B) and the immune system (e.g., RIG-I-like receptor signaling pathway) (Fig. 10C). Interestingly, we also found a different pathway for bacterial invasion of epithelial cells which could putatively increase the expression of SipD and SipB in the Salmonella infections pathway, and increased the IpaB expression in the Shigella infections pathway in HM (Fig. 11A.B). The CAZymes analysis showed that longer corn storage times resulted in higher levels of the following subsystems when compared with the shorter storage treatments; cellulose_synthase, chitin_oligosaccharide synthase, chitin_synthase, xylanase, arabinanase and glucosaminidase (Table S4).

## Discussion

### The effect of storage on corn properties

Compared to freshly harvested grain, a change in protein digestibility was reported by Paraginski et al. [5] and Ramchandran et al. [28], potentially due to protein and starch solubility. One of the most sensitive indices of the aging in grain is the change in pasting properties [29, 30] which is related to the granule size and soluble materials of starch [31]. As storage duration increased, peak viscosity, final viscosity and setback decreased which were in agreement with previous studies [32, 33]. This is attributed to the formation of a complex between amylose and lipids and thereby restriction of granular swelling.

Dietary sugars (glucose and fructose) that escape absorption in the mammalian intestine and reach the microbiota disrupt colonization by beneficial microbes [34]. In the current study we observed an increase in total soluble sugars and reductions in glucose and fructose in the stored corn which were in concordance with the findings of Paraginski [5]. We observed that higher oligosaccharide and lower monosaccharide contents in stored corn could benefit gut health [35].

### Differentially expressed proteins

We studied the effect of corn storage and carbohydrate composition as storage is known to exhibit strong effects on starch biosynthesis [36]. Li et al. [37] reported that starch retrogradation is mainly caused by the aggregation of amylose and occurs during the early stage of storage. 1,4-α-glucan-branching enzyme activity catalyzes ADP-glucose into amylopectin, inhibiting starch retrogradation [37, 38]. The upregulation of the 1,4-α-glucan-branching enzyme in TM suggested a decrease in the amount of amylose and an increase in amylopectin which could increase the water-insolubility of starch and protein [39]. During storage, respiratory and metabolic events continue. Pyruvate, phosphate dikinase and phosphoenolpyruvate carboxylase family proteins play key roles in CO_2_ transport and fixation, respectively [40]. Higher activities of these two enzymes under TM suggested a higher synthetic ratio of starch when carbon gain exceeds the carbon demands for growth (accumulation) [41].

In addition to storage, the upregulation of cellulose synthase, a catalytic subunit could catalyze secondary cell wall biogenesis and improve the ability to defend against bacteria and fungi. The significant upregulation of dirigent proteins, which are thought to play important roles in plant secondary metabolism and are the first intermediates for lignan biosynthesis, was observed in stored corn [42]. Lignans are known to form building blocks for the formation of lignin in the plant cell wall and are metabolized by intestinal bacteria and exhibit strong antioxidant and bioactive properties such as enzymes and protein synthesis, cell proliferation, growth factor action and cell differentiation [43].

The aging process affects storage proteins, proteins related to cell growth and division, and cell defense [44]. During storage, grain seeds could mobilize the storage substances in a timely and efficient manner to obtain a sufficient carbon source and energy supply for germination. The upregulation of Legumin-1 in stored corn suggested that germination activity could be improved with storage. Protein oxidation (carbonylation) and reduced translation always occur during aging [45].

### Functional annotation of identified proteins

In the current study, the molecular function of most proteins was related to the activity of peptidase. Peptidases are enzymes that hydrolyze peptide bonds in proteins and peptides and release amino acids, peptides, and proteins from larger peptides and proteins [46]. From the cellular process analysis, storage duration had a major impact on protein homeostasis and induced proteins involved in metabolic processes (mainly the protein catabolic process and amino acid biosynthetic process). For dried stored corn seed, free amino acids in grain increased with storage and there is a decrease in the lower molecular weight peptides and an increase in the higher molecular weight peptides [39]. The texture changes in stored grains are the consequence of modifications by of carbohydrate polymers by component polysaccharides via hydrolytic enzyme activity. The chemical and proteomic analysis results suggest that storage probably induced an increase in large molecular weight starch and protein and produced modifications in solubility. This could lead to lower starch pasting properties and higher concentrations of beneficial oligosaccharides which could affect animal health.

### Whole genome shotgun metagenome analysis

In the current study, increased storage time resulted in increased AME and AMEn, is similar to the results reported by Fuente et al. [47]. The intestinal flora has been recently proposed to affect body weight and energy homeostasis. The results from our RDA analysis showed a strong correlation between AME and *Acidaminococcus* sp. which has an effect on growth through the fermentation of glutamate [48]. Mucin is secreted by goblet cells along the villi of the epithelium. The present study has indicated that the number of goblet cells was decreased under the TM corn treatment. Goblet cell function revealed the metabolic and inflammatory phenotypes to the intestinal microbiota. Johansson et al. [49] reported that more anti-nutritional factors led to epithelial cell apoptosis along the villi and crypts, fusion of villi and more and larger goblet cells. On the other hand, the regeneration of epithelial goblet cells is consistent with *Acidaminococcus* colonization [50].

The lower enrichment of Firmicutes under the TM treatment was in concordance with the result of Stanley et al. [51] who reported that a class of Firmicutes was negatively correlated with poultry energy utilization. Members of the genus *Bifidobacteria* are the main gut microbiota recognized as being beneficial for health. Two months stored corn significantly upregulated the enrichment of *Bifidobacteria* which impacts positively the host and the target of prebiotic functional foods and supplements [52]. At the genus level, we found that TM treatment had lower enrichment in *eggerthella*. *Eggerthella* have been implicated as a cause of ulcerative colitis, liver and anal abscesses and systemic bacteremia [53].

*Bacteroides fragilis*, a gram-negative, obligately anaerobic bacterium that was negatively correlated with AME_n_ [54], the present study found that higher enrichment of *Bacteroides fragilis* in HM might reduce the host immune response toward pathogenic bacteria by suppressing inflammatory pathways. Enterotoxin production by *B. fragilis* was identified and subsequently found to produce severe diarrheal disease in several intact animals [55]. Strauss et al. [56] reported that *Fusobacterium* isolated from IBD patients is a bacterial species associated with inflammatory disease. From the LEfSe analysis, newly harvested corn treatment led to higher relative abundance of *Hungatella hathewayi*, *Bacteroides fragilis* and *Fusobacterium* which might colonize the gut and produce enterotoxins to induce the diarrheal response and be the major reason for the “newly harvested grain problem”. *Hungatella hathewayi* can cause a wide spectrum of illnesses ranging from mild diarrhea to pseudomembranous colitis [57]. The TM treatment seemed to enrich in butyrate-producing bacteria such as *Alistipes* that typically play anti-inflammatory roles. Thus, prolonging the storage time could improve gut health by decreasing the abundance of harmful microorganisms in the intestine.

### Microbial gene functional diversity

The core functions of the intestinal microorganisms include pathways associated with carbohydrate and amino acid fermentation. It has been demonstrated that the cecal microbiome was enriched in genes involved in carbohydrate metabolism plus the metabolism of galactose and fructose from the stored corn. Several dietary components, especially polysaccharides such as inulin, fructooligosaccharides or xylan are not modified or absorbed in the intestine and are considered as prebiotic promoting the growth of beneficial bacteria [58]. These microbes essentially assist the host in deriving maximum nutritional value from the components of the diet. Microbial fermentation of indigestible polysaccharides results in the production of SCFAs which are linked to the immune system and enterocyte development [59].

Glycoside hydrolases (GHs) are essential enzymes required for the breakdown of polysaccharides. Polysaccharide degrading enzymes contribute to metabolic energy. The higher activities of GHs found in TM were associated with the better utilization of energy such as AME and AMEn and related to the production of SCFAs. The distal gut microbiome provides the host with the capacity to degrade these glycans. In addition, many members of the microbiota possess the ability to synthesize new polysaccharides de novo. Bacterial production of capsular polysaccharides is associated with increased resistance to phages, complements, and antimicrobial peptides [54]. *Bacteroides* spp. are known to breakdown a wide variety of otherwise indigestible dietary plant polysaccharides (eg., amylose, amylopectin and pullulan) [60], while proteomic analysis showed that higher amylopectin synthetase activity with the storage of corn might contribute to increasing the abundance of *Bacteroides* under the TM and stimulate glycan biosynthesis and metabolism. Glycosyltransferases (GTs) are enzymes that catalyze the formation of the glycosidic linkage to form a glycoside and involve in the synthesis of glycosphinogolipids [61]. Glycosphingolipids are building blocks of the plasma membrane that determine lipid rafts and are involved in cell functions such as proliferation, apoptosis and embryogenesis [62]. Higher enrichment in GTs potentially improved the synthesis of glycosphingolipids for TM suggested that stored corn fed to broilers could improve the function of epithelial cells.

Beyond digestion and metabolism, the microbiota contributes to the development and maintenance of the intestinal epithelial barrier, development of the immune system, and competition with pathogenic microorganisms. Among these systems, RIG-I-like receptor signaling plays a major role in pathogen sensing of RNA viral infection to initiate and modulate antiviral immunity [63], and it was influenced by the treatments. Salmonella infections have the capacity to modulate cellular functions and induce profuse actin cytoskeleton rearrangements and nuclear responses that ultimately lead to bacterial uptake and the production of proinflammatory cytokines [64]. Shigellae are the etiological agents of bacillary dysentery and the acute form of diarrhea accompanied by blood and mucus [65]. Newly harvested corn increases the expression of SipD and SipB in the Salmonella infection pathway and increases IpaB expression in the Shigella infections pathway. SipB and IpaB apparently bind and activate caspase-1 and result in the stimulation of an unconventional form of programmed cell death with features of necrosis, which was in accordance with the intestinal morphological results. Consequently, a higher possibility of Salmonella and Shigellae infections could potentially occur in broiler chickens fed a diet made from newly harvested corn, which could result in diarrhea and intestinal epithelial cell death.

## Conclusion

Natural aging of newly harvested corn is a complex process. We used proteomics to identify 26 proteins that could be responsible for the synthesis of large molecules and change the solubility of starch and protein, as well as soluble sugars and decrease pasting properties. Compared with newly harvested corn, two-month-stored corn appears to have a decreased abundance of harmful bacteria such as *Hungatella hathewayi* and *Bacteroides fragilis*, with an enrichment of butyrate-producing bacteria such as *Alistipes* in the guts of broiler chickens. The functional roles of these host bacteria coupled with the low risk of diarrhea and higher utilization of energy are clues to the underlying mechanisms of the “new grain problem”.

## Supplementary Information

**Additional file 1: Table S1.** The Gradient elution procedure in LC-MS/MS analysis. **Table S2.** Composition of the basal diets (air-dry basis). **Table S3.** Composition of the Metabolic diets (air-dry basis). **Table S4.** CAZymes analysis. **Fig. S1.** Venn diagram showing the overlap of protein identities in two stored period of freshly harvested corn for (B1) half month storage and B2 two months storage. **Fig. S2.** The proportion of the differentially expressed protein categorized by KEGG pathway. **Fig. S3.** Influence of corn storage on duodenal villi height and crypt depth (A) and the ratio of villi and crypt (B) for broiler chickens. **Fig. S4.** Venn graph. **Fig. S5.** Relative abundance of annotated significant different species (Top 10) in cecal microbiota of broilers.

## Data Availability

The datasets used and /or analyzed during the current study are available from the corresponding author on request.

## Abbreviations

HM: Half months stored corn
TM: Two months stored corn
AME: Apparent Metabolisable Energy
AME_n_: Nitrogen-corrected Apparent Metabolisable Energy
SCFA: Short-Chain Fatty Acids
DEPs: Differentially Expressed Proteins
IBD: Inflammatory Bowel Disease
RVA: Rapid Visco-Analyzer
CAZy: Carbohydrate enzyme
GHs: Glycoside hydrolases
HPLC: High Performance Liquid Chromatography
LDA: Linear Discriminant Analysis
RDA: Redundancy analysis
