# Supplementary material for: Insights into the proteomic profile of newly harvested corn and metagenomic analysis of the broiler intestinal microbiota
Source: J Anim Sci Biotechnol. 2022 Feb 9;13:26. doi: 10.1186/s40104-021-00656-1 (PMC8827200; doi:10.1186/s40104-021-00656-1)
Supplement: Supplementary file 1 — Additional file 1: Table S1. The Gradient elution procedure in LC-MS/MS analysis. Table S2. Composition of the basal diets (air-dry basis). Table S3. Composition of the Metabolic diets (air-dry basis). Table S4. CAZymes analysis. Fig. S1. Venn diagram showing the overlap of protein identities in two stored period of freshly harvested corn for (B1) half month storage and B2 two months storage. Fig. S2. The proportion of the differentially expressed protein categorized by KEGG pathway. Fig. S3. Influence of corn storage on duodenal villi height and crypt depth (A) and the ratio of villi and crypt (B) for broiler chickens. Fig. S4. Venn graph. Fig. S5. Relative abundance of annotated significant different species (Top 10) in cecal microbiota of broilers. [file 40104_2021_656_MOESM1_ESM.docx]

| Table S1 The Gradient elution procedure in LC-MS/MS analysis | | | |
| --- | --- | --- | --- |
| Time, min | A | B | Speed, nL/min |
| 0 | 94% | 6% | 600 |
| 8 | 91% | 9% | 600 |
| 24 | 86% | 14% | 600 |
| 60 | 70% | 30% | 600 |
| 75 | 60% | 40% | 600 |
| 78 | 5% | 95% | 600 |
| 85 | 5% | 95% | 600 |
| 86 | 94% | 6% | 600 |
| 90 | 94% | 6% | 600 |

| Table S2 Composition of the basal diets (air-dry basis) | | | |
| --- | --- | --- | --- |
| Ingredients | Content, % | Calculated nutrient content | |
| Corn | 58.00 | AME, Mcal/kg | 2.90 |
| Soybean meal | 35.00 | CP, % | 21.30 |
| Soybean oil | 2.75 | Ca, % | 1.00 |
| DL-Met | 0.20 | Lys, % | 1.20 |
| L-Lys.HCL | 0.02 | Met, % | 0.50 |
| Limestone | 1.25 | Met+Cys, % | 0.85 |
| CaHPO_4_ | 2.00 | Thr, % | 0.85 |
| NaCl | 0.30 | AP, % | 0.47 |
| Trace mineral premix^1）^ | 0.20 |  |  |
| Trace Vitamine premix^2）^ | 0.03 |  |  |
| 50% Choline chloride | 0.25 |  |  |
| Total | 100 |  |  |

^1）^The trace mineral premix provided the following per kg of diets: Cu 8mg, Zn 75mg, Fe 80mg, Mn 100mg, Se 0.15mg, I 0.35mg

^2）^The vitamin premix provided the following per kg of diets：VA 12500 IU, VD_3_ 2500 IU, VE 18.75mg, VK_3_ 2.65mg，VB_2_ 6mg, VB_12_ 0.025mg, biotin 0.0325mg, folic acid 1.25mg, pantothenic acid 12mg, nicotinic acid 50mg.

| Table S3 Composition of the Metabolic diets (air-dry basis) | | |
| --- | --- | --- |
| Ingredients | | Content, % |
| Corn | | 96.00 |
| CaHPO_4_ | | 1.80 |
| Limestone | | 1.13 |
| NaCl | | 0.35 |
| Trace mineral premix^1）^ | | 0.02 |
| Trace Vitamine premix^2）^ | | 0.20 |
| Titanium dioxide | | 0.50 |
| Total |  | 100.00 |

^1）^The trace mineral premix provided the following per kg of diets: Cu 8mg, Zn 75mg, Fe 80mg, Mn 100mg, Se 0.15mg, I 0.35mg

^2）^The vitamin premix provided the following per kg of diets：VA 12500 IU, VD_3_ 2500 IU, VE 18.75mg, VK_3_ 2.65mg，VB_2_ 6mg, VB_12_ 0.025mg, biotin 0.0325mg, folic acid 1.25mg, pantothenic acid 12mg, nicotinic acid 50mg.

| Table S4 CAZymes analysis | | | | |
| --- | --- | --- | --- | --- |
| Taxa | HM | TM | *P*-value | q-value |
| GH59 | 1.7E-06 | 7.30E-05 | <0.001 | <0.001 |
| CE7 | 1.0E-06 | 1.58E-04 | 0.001 | 0.024 |
| GT14 | 4.6E-06 | 5.54E-05 | 0.001 | 0.032 |
| GH106 | 2.0E-06 | 2.71E-04 | 0.002 | 0.036 |
| GH109 | 7.8E-05 | 3.63E-04 | 0.002 | 0.038 |
| GH115 | 5.7E-05 | 3.23E-04 | 0.003 | 0.041 |
| GH73 | 4.7E-04 | 3.13E-04 | 0.004 | 0.042 |
| PL10 | 1.4E-06 | 2.81E-04 | 0.004 | 0.043 |
| GH63 | 4.8E-05 | 1.86E-04 | 0.005 | 0.043 |
| PL17 | 1.3E-05 | 1.20E-06 | 0.005 | 0.044 |
| CBM57 | 1.4E-06 | 2.07E-04 | 0.006 | 0.044 |
| GT30 | 6.0E-05 | 2.67E-04 | 0.006 | 0.044 |
| GH67 | 1.2E-05 | 7.65E-05 | 0.007 | 0.045 |
| CE12 | 7.0E-05 | 4.25E-04 | 0.007 | 0.045 |
| GH108 | 1.5E-05 | 1.78E-04 | 0.008 | 0.045 |
| CBM58 | 6.1E-06 | 9.19E-05 | 0.008 | 0.045 |
| GH55 | 8.0E-06 | 2.30E-05 | 0.009 | 0.045 |
| CBM51 | 3.8E-05 | 7.08E-05 | 0.009 | 0.045 |
| GH28 | 4.9E-04 | 1.77E-03 | 0.010 | 0.046 |
| GH105 | 2.4E-04 | 1.06E-03 | 0.010 | 0.046 |
| GH89 | 5.2E-05 | 2.59E-04 | 0.011 | 0.046 |
| CE8 | 9.0E-05 | 3.75E-04 | 0.011 | 0.046 |
| GH24 | 7.0E-05 | 2.09E-04 | 0.012 | 0.046 |
| GH97 | 1.2E-04 | 7.23E-04 | 0.012 | 0.046 |
| PL11 | 2.9E-05 | 2.06E-04 | 0.013 | 0.046 |
| GH5 | 1.3E-04 | 3.29E-04 | 0.013 | 0.046 |
| PL6 | 0.0E+00 | 2.23E-07 | 0.014 | 0.046 |
| GH78 | 2.8E-04 | 5.65E-04 | 0.014 | 0.046 |
| CE14 | 6.2E-05 | 2.01E-05 | 0.015 | 0.048 |
| PL1 | 4.8E-05 | 1.58E-04 | 0.016 | 0.048 |
| GH92 | 2.2E-04 | 1.23E-03 | 0.016 | 0.048 |
| GH43 | 1.1E-03 | 2.94E-03 | 0.017 | 0.048 |
| GH66 | 4.9E-06 | 1.59E-05 | 0.017 | 0.048 |
| CBM59 | 0.0E+00 | 4.85E-06 | 0.018 | 0.048 |
| GT9 | 1.2E-04 | 3.59E-04 | 0.018 | 0.048 |
| CBM3 | 1.6E-06 | 6.01E-06 | 0.019 | 0.048 |
| GT34 | 4.7E-07 | 2.37E-05 | 0.019 | 0.048 |
| GH47 | 5.9E-05 | 2.22E-05 | 0.020 | 0.048 |
| GH84 | 7.4E-05 | 1.63E-04 | 0.020 | 0.048 |
| Others | 9.7E-01 | 9.48E-01 | 0.021 | 0.048 |
| GH30 | 7.8E-05 | 1.84E-04 | 0.022 | 0.048 |
| CE15 | 3.6E-05 | 1.15E-04 | 0.022 | 0.048 |
| GT66 | 2.5E-04 | 1.55E-04 | 0.023 | 0.048 |
| GH132 | 0.0E+00 | 3.16E-07 | 0.023 | 0.048 |
| GH15 | 3.0E-06 | 5.93E-05 | 0.024 | 0.048 |
| CBM62 | 3.1E-05 | 1.35E-04 | 0.024 | 0.048 |
| GH93 | 1.3E-06 | 6.41E-06 | 0.025 | 0.048 |
| CBM20 | 4.6E-05 | 1.31E-04 | 0.025 | 0.048 |
| GT3 | 1.7E-05 | 1.04E-04 | 0.026 | 0.048 |
| GH9 | 2.8E-05 | 1.01E-04 | 0.026 | 0.048 |
| GH127 | 2.2E-04 | 3.61E-04 | 0.027 | 0.048 |
| GH117 | 5.2E-05 | 1.81E-04 | 0.027 | 0.048 |
| GT20 | 1.2E-05 | 7.13E-05 | 0.028 | 0.048 |
| GH85 | 2.8E-05 | 1.10E-04 | 0.028 | 0.048 |
| GT41 | 6.0E-07 | 1.61E-05 | 0.029 | 0.048 |
| GT19 | 4.1E-05 | 1.40E-04 | 0.029 | 0.048 |
| CBM14 | 6.5E-07 | 6.39E-06 | 0.030 | 0.048 |
| GH58 | 4.9E-07 | 2.52E-06 | 0.030 | 0.048 |
| GH16 | 1.7E-04 | 5.23E-04 | 0.031 | 0.048 |
| GH20 | 4.4E-04 | 1.06E-03 | 0.031 | 0.048 |
| CBM32 | 3.1E-04 | 4.75E-04 | 0.032 | 0.048 |
| GH116 | 1.7E-05 | 3.78E-05 | 0.032 | 0.048 |
| GH32 | 7.7E-04 | 4.12E-04 | 0.033 | 0.048 |
| GH53 | 3.5E-05 | 1.02E-04 | 0.034 | 0.049 |
| CBM66 | 1.0E-04 | 1.21E-05 | 0.035 | 0.049 |
| CE9 | 3.9E-04 | 4.62E-04 | 0.038 | 0.049 |
| GH130 | 1.5E-04 | 3.26E-04 | 0.036 | 0.049 |
| GH2 | 1.7E-03 | 3.03E-03 | 0.036 | 0.049 |
| GH72 | 2.2E-04 | 3.19E-04 | 0.037 | 0.049 |
| GH79 | 2.6E-05 | 5.20E-06 | 0.039 | 0.049 |
| GH88 | 2.2E-04 | 4.14E-04 | 0.035 | 0.049 |
| GH95 | 3.0E-04 | 6.06E-04 | 0.039 | 0.049 |
| GT32 | 8.0E-05 | 1.63E-04 | 0.040 | 0.049 |
| GT73 | 3.8E-07 | 2.61E-06 | 0.038 | 0.049 |
| PL22 | 1.4E-05 | 6.01E-05 | 0.037 | 0.049 |
| GH68 | 3.8E-06 | 4.49E-07 | 0.041 | 0.049 |


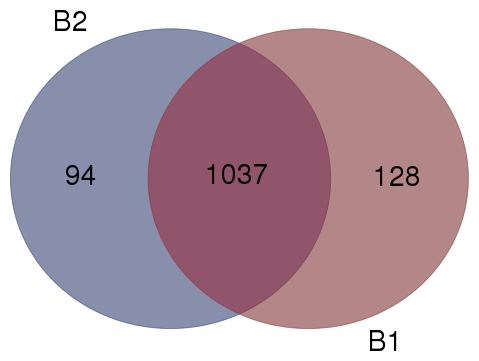


HM

TM

Fig. S1 Venn diagram showing the overlap of protein identities in two stored period of freshly harvested corn for (B1) half month storage and B2 two months storage.

Fig. S2 The proportion of the differentially expressed protein categorized by KEGG pathway.

Fig. S3 Influence of corn storage on duodenal villi height and crypt depth (A) and the ratio of villi and crypt (B) for broiler chickens. HM: half month storage corn; TM: two months storage corn.


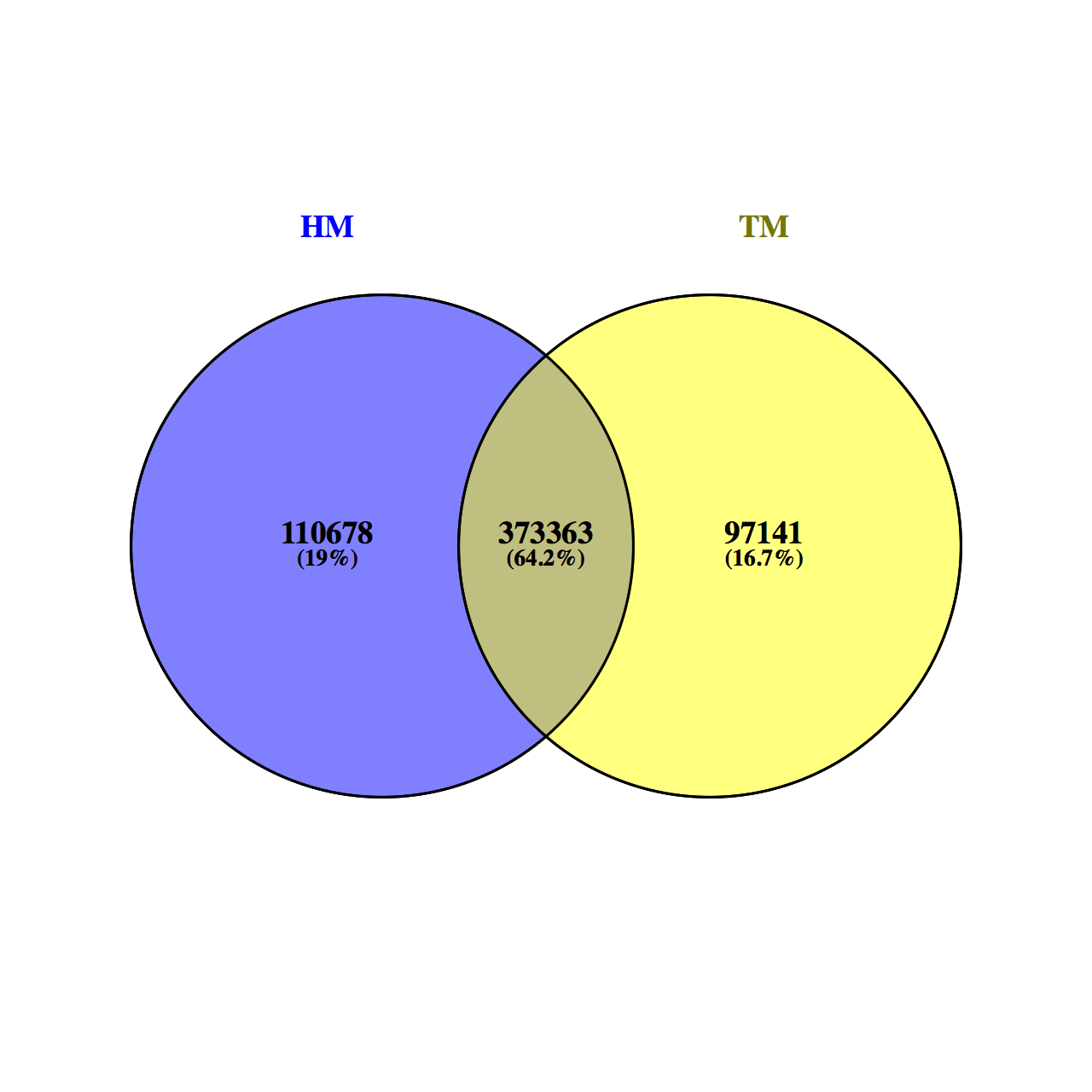


Fig. S4 Venn graph

(HM: half month storage corn; TM: two months storage corn)

Fig. S5 Relative abundance of annotated significant different species (Top 10) in cecal microbiota of broilers. (HM, half months stored corn; TM, two months stored corn)
